# Optimizing and evaluating PCR-based pooled screening during COVID-19 pandemics

**DOI:** 10.1038/s41598-021-01065-0

**Published:** 2021-11-02

**Authors:** Jiali Yu, Yiduo Huang, Zuo-Jun Shen

**Affiliations:** 1 Tsinghua-Berkeley Shenzhen Institute (TBSI), Tsinghua University, Shenzhen, China; 2 Department of Civil and Environmental Engineering, University of California Berkeley, Berkeley, CA USA; 3 College of Engineering, University of California Berkeley, Berkeley, CA USA; 4 Faculty of Engineering and Faculty of Business and Economics, University of Hong Kong, Hong Kong, China

**Keywords:** Epidemiology, Population screening

## Abstract

Population screening played a substantial role in safely reopening the economy and avoiding new outbreaks of COVID-19. PCR-based pooled screening makes it possible to test the population with limited resources by pooling multiple individual samples. Our study compared different population-wide screening methods as transmission-mitigating interventions, including pooled PCR, individual PCR, and antigen screening. Incorporating testing-isolation process and individual-level viral load trajectories into an epidemic model, we further studied the impacts of testing-isolation on test sensitivities. Results show that the testing-isolation process could maintain a stable test sensitivity during the outbreak by removing most infected individuals, especially during the epidemic decline. Moreover, we compared the efficiency, accuracy, and cost of different screening methods during the pandemic. Our results show that PCR-based pooled screening is cost-effective in reversing the pandemic at low prevalence. When the prevalence is high, PCR-based pooled screening may not stop the outbreak. In contrast, antigen screening with sufficient frequency could reverse the epidemic, despite the high cost and the large numbers of false positives in the screening process.

## Introduction

COVID-19 outbreaks have caused enormous economic losses and have posed health risks, particularly among disadvantaged populations. Testing plays a critical role in containing these outbreaks and lifting containment measures. Tests for COVID-19 are typically divided into two types according to their goals and focus on diagnostic tests and screening tests. Diagnostic tests which prioritize symptomatic individuals are responsible for supporting individual medical treatments. These tests require high testing sensitivity, and their results must be interpreted along with the exposure history and symptoms of patients. In contrast, the goal of screening is to identify and help isolate pre-symptomatic and asymptomatic patients who may unwittingly transmit the virus^1^. A growing number of countries, including China, Slovakia, Iceland, and the UK, have conducted population screening to mitigate COVID-19 spread using PCR-based pooled or antigen tests^1^. However, questions have arisen, specifically, given different screening strategies and technologies, (1) how we can evaluate their cost-effectiveness, and (2) what strategies we should adopt to stop the spread of COVID-19.

After the pandemic began, PCR tests, which directly detect the genetic material of the severe acute respiratory syndrome-coronavirus-2 (SARS-CoV-2), were rapidly developed following the publication of the reference genome^1^. However , the PCR test is a time-consuming process that requires sophisticated laboratory equipment and highly trained technicians. Therefore, PCR-based pooled testing (also called Dorfman pooling), which tests samples drawn from multiple individuals as a pooled sample, has been proposed to test more people with existing equipment and technicians^2,3^. If a pool tests negative, every sample in the pool is considered negative. Otherwise, all samples need to be re-tested individually because at least one individual in this pool is infected. The practice of pooled screening has shown that Dorfman pooling can significantly increase testing efficiency despite the loss of accuracy in the early stage of transmission. For example, China has adopted Dorfman pooling as a regular tool to screen populations with no symptoms until community transmission ends. Shenzhen, a city of more than 17 million people, conducted two rounds of city-wide population screening, five rounds of screening in Yantian District, and four rounds of screening in Bao’an District by PCR-based Dorfman pooling between May and June 2021 to end the resurgence of COVID-19. The fourth round of screening in Bao’an involved 815 sample collection sites and 5228 medical personnel. A total of 5,195,328 people were tested, with no confirmed cases within 44 h from June 28 to 30, 2021. By the end of June 2021, the daily PCR testing capacity of Shenzhen had reached 550,000 single tubes. By pooling ten samples in one assay, Shenzhen can test 4 million people per day^4,5^.

Compared to PCR tests, antigen tests with lower sensitivity take longer to develop but provide point-of-care test results and can be quickly scaled to large quantities. For example, Slovakia conducted two rounds of population screening using rapid antigen tests in late 2020. Combined with strict social distancing orders, infection prevalence declined by about 80%^6^.

Although antigen tests offer an alternative way to increase testing capacity, we focus on studying the PCR-based pooled screening in a real-world context, as PCR tests can be quickly developed and scaled up through existing infrastructure in the early stage of an epidemic outbreak. Furthermore, establishing the capability of PCR-based pooled screening expedites responses to the current COVID-19 pandemic and will play a central role in preparation for future pandemics.

A recent review of scaling testing highlighted the role of population screening in combating the COVID-19 pandemic^1^. Compared to the Dorfman pooling, more sophisticated testing algorithms that may be more efficient have been proposed in the literature, including hypercube pooling testing^7^, non-random pool allocations^8^, multi-stage hierarchical pool testing^9^, and combinatorial non-hierarchical pool testing^10,11^. However, such sophisticated testing algorithms can be difficult to implement, especially in population screening. Therefore, the Dorfman pooling and antigen tests remain the most widely used methods in population screening because of their simplicity and effectiveness. Although the literature on Dorfman pooling and antigen testing has grown rapidly, the cost-effectiveness of different testing technologies and strategies under fast-changing epidemic dynamics remains unclear. Within the framework of simple Dorfman pooling, most recent studies have focused on the optimal design of pool size, modifications of the pooling workflows, and corresponding laboratory validation^7,8,10,12–16^. The efficiency, accuracy^7,12,14^, and cost-effectiveness^17^ of pooled testing were evaluated under a set of static points of prevalence. These studies often assumed a fixed value for test sensitivity in their models, but experimental studies showed that pool size and prevalence had significant impacts on test sensitivity in many pooled algorithms^7,8,14,18–28^. Braul et al. ^29^ and Cleary et al.^10^ bridged this gap by considering diluted sensitivities and viral load kinetics of SARS-CoV-2. Cleary et al. studied the design and dilution effects of pooled testing from the new perspective of population-level viral load distribution during the epidemic^9^. This new perspective is valuable in enlightening the investigation of many important issues during the pandemic, for example, the estimation of epidemic dynamics^10,30^ and the evaluation of large-scale screening. In addition, statistical models were developed to estimate the prevalence and individual-level risk of infection based on pooled testing outcomes^10,31–38^. Digital technologies were proposed to enhance the effectiveness of population screening^39–47^. Studies also revealed that repeated PCR testing, antigen testing, and household-pooled testing could effectively suppress outbreaks^6,8,48,49^.

As implementing PCR-based pooled screening as a large-scale intervention has not been comprehensively evaluated, we focused on comparing PCR-based pooled screening with different population screening methods as the intervention tool under a dynamic transmission model. Incorporating the testing-isolation process and individual-level viral load trajectories into an epidemic model, we studied the interaction between screening and population-level viral load distribution. Our results show that the testing-isolation process could maintain a stable test sensitivity during the outbreak by removing most infected individuals. Moreover, we compared PCR-based pooled screening with individual PCR and antigen screening from the perspectives of containment effect, accuracy, and cost-effectiveness. Due to the systematic error of hierarchical operation and dilution effects, the sensitivity of pooled PCR testing cannot surpass individual PCR testing. Despite the sensitivity loss due to hierarchical operation and dilution effects, pooled PCR could facilitate the high-frequency population screening at low prevalence, reducing the total number of infections. Moreover, pooled screening could reduce false-positive results by testing positive samples twice, avoiding unnecessary confirmatory tests or isolation of health people. Finally, our results indicate that PCR-based pooled screening is cost-effective for containing the outbreak at low prevalence.

## Methods

We defined the population-level viral load distribution as all unidentified infected individuals, as isolated patients have been removed from the transmission chain. First, we studied the interaction of screening and population-level viral load distribution by incorporating the testing-isolation protocol and individual-level viral load trajectories into an epidemic transmission model. Then, we evaluated and compared PCR-based pooled screening with other different population screening methods during the pandemic.

### Investigating the interaction between viral load distributions and screening sensitivities

On the one hand, population-level viral load distribution affects the sensitivities and the design of pooled screening. On the other hand, the testing-isolation process has a great impact on the viral load distribution. We simulated epidemic outbreaks incorporating individual-level viral loads and testing-isolation process to capture this complicated interaction. First of all, we adopted the viral load kinetic model proposed by Cleary et al. to generate the individual-level viral load trajectories throughout the pandemic^10^. Parameters for the viral load trajectories were estimated based on pharyngeal swab samples (see Table S1 in “Supplementary Materials” for the overview of viral kinetic parameters)^50^.

Then, we compared the test sensitivities by simulating the pandemic with/without the testing-isolation process. On a specific day in one simulation, we can estimate the sensitivities by simulating random pooled tests. We randomly assigned participants into groups, and the pooled viral load of one group was the average viral load of samples in that group. To evaluate the policies and find the optimal pool size, we considered three sensitivities: the sensitivity of PCR tests using the individual sample, $S_{ei} \left( t \right): = \frac{positive\;infected\;individual\;samples}{{infected\;individual\;samples}}$, the sensitivity of PCR tests using pooled samples, $S_{ep} \left( {t,n} \right): = \frac{positive\;infected\;pooled\;samples}{{infected\;pooled\;samples}}$, and the overall sensitivity of two-stage pooled testing, $S_{ed} \left( {t,n} \right): = \frac{positive\;infected\;test\;participants}{{infected\;test\;participants}}$, where $t$ (days) is the time since the beginning of the pandemic, $n$ is the pool size. To reduce the stochastic error of these sensitivity estimations, we ran the simulation many times and used the locally weighted scatterplot smoothing (LOWESS) regression to fit ${S}_{ei}(t)$, ${S}_{ep}(t,n)$, and ${S}_{ed}(t,n)$ numerically.

### Optimal pool size design

Screening during a pandemic aims to identify and remove infected patients from the transmission chain as soon as possible. We measured the overall efficiency and effectiveness of screening by the expected number of tests required to identify an infected patient, defined as the cost-performance ratio (CPR). Note that our setting of CPR optimization only considers the test effectiveness of a given test capacity, while the administrative cost will be discussed later. According to its definition, $CPR\left( {t,n} \right)$ is given by:

1 $$CPR\left( {t,n} \right) = \left\{ {\begin{array}{*{20}l} {\frac{1}{{S_{ei} (t)*p}},} \hfill & {n = 1,} \hfill \\ {\frac{1}{{S_{ed} (t,n)*p}}\left\{ {\frac{1}{n} + S_{ep} (t,n) - [S_{ep} (t,n) + S_{p} - 1]\left( {1 - p} \right)^{n} } \right\},} \hfill & {n > 1.} \hfill \\ \end{array} } \right.$$

where ${S}_{p}$ represents the test specificity (true negative rate) for the PCR test, $p$ is the prevalence. $S_{ed} (t,n)$, $S_{ep} (t,n)$, and $S_{ei} (t)$ represent the sensitivity of overall Dorfman pooling, the first-stage pooling, and individual PCR testing, respectively.

The optimal pool size, ${n}^{*}(t)$, at time $t$, was calculated by ${n}^{*}(t)=\underset{n\in \mathcal{N}}{\mathrm{argmax}}CPR(t,n)$, where $\mathcal{N}$ is the candidate set for the pool size. We use a fixed set of candidate pool sizes ${\mathcal{N}}: =$ (1, 4–10, 15, 20, 25, 30) to ensure that the pool size is stable across a range of prevalence rates, avoiding a frequent change of pool sizes. This range of candidate pool sizes is based on an experimental study that evaluated the effect of pooling samples (a range of pool sizes, from 4 to 30 samples per pool) ^3^.

### Epidemic model

**Figure 1:**
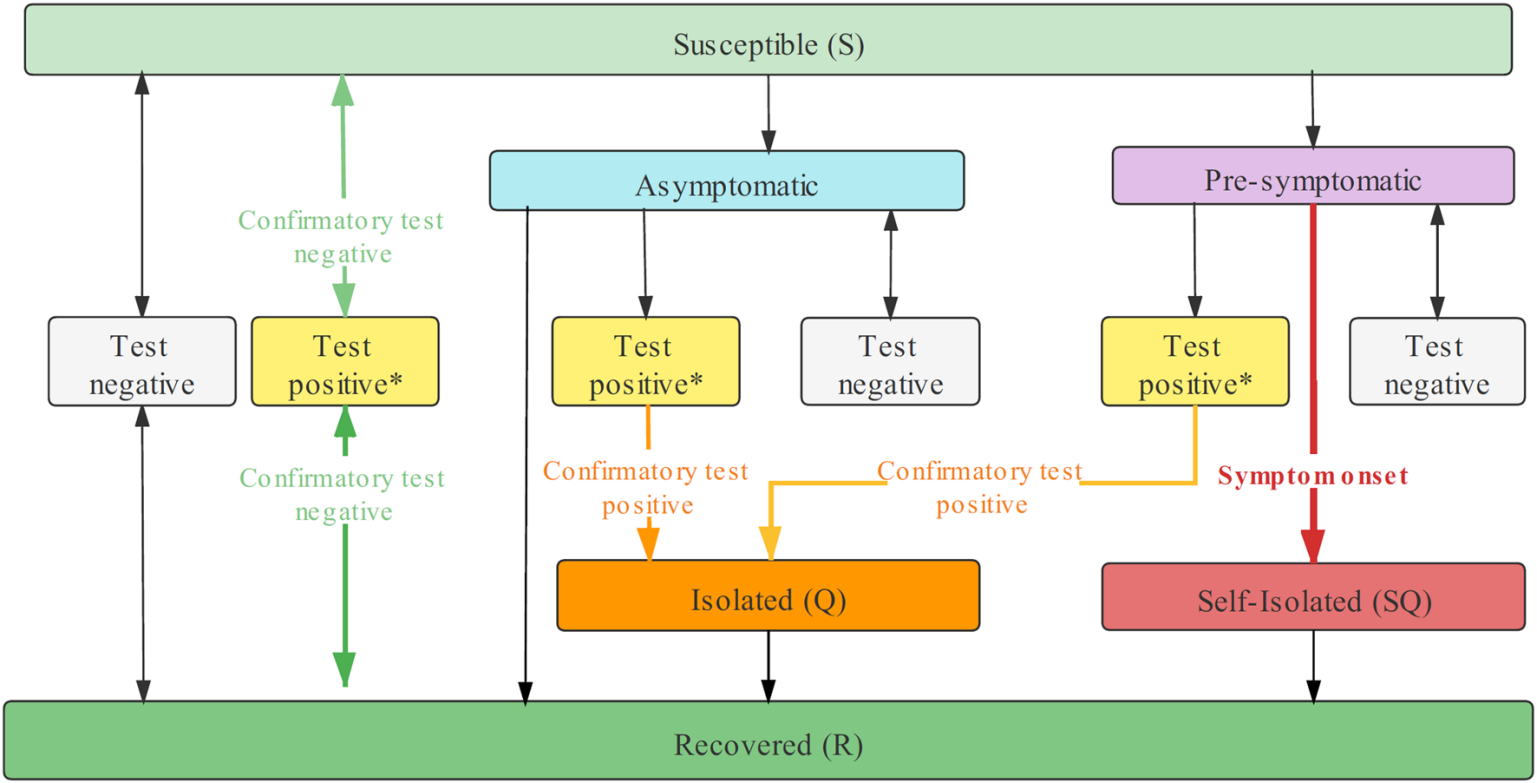
The epidemic model with testing-isolation protocols. Susceptible individuals are infected through contact with infected individuals. They will go through an incubation period and either remain asymptomatic or develop symptoms. Symptomatic patients will do self-isolation after developing symptoms. All participants, except isolated and self-isolated individuals, will take part in the screening. If screening results are positive, people need another confirmatory test to exclude false-positive results. If confirmatory tests have positive results, this individual will be isolated. If screening gives a negative result, participants will remain in their previous compartments. All infected individuals will finally recover.

We incorporated individual-level viral load trajectories and testing-isolation into a SARS-CoV-2 transmission model where we track discrete individuals who are Susceptible (S), Infected (I), Isolated (Q), Self-Isolated (SQ), and Recovered (R) every day. The epidemic dynamics and the testing-isolation process is shown in Fig. 1.

At the beginning of the simulation, 50 infected individuals (i.e., ${I}_{0}=50$) who remained in the incubation period were mixed in a total population of *N* = 100,000. The remaining populations are susceptible ($S=N-{I}_{0}$). At each time step, susceptible individuals were infected through contact with unidentified infected individuals (S → I). Newly infected individuals were randomly sampled from susceptible populations and assigned the current time as their infection time. Later, the daily viral load was generated for each infected individual according to their infection time and current time based on the viral kinetic model proposed by Clearly et al.^10^. Infected individuals would isolate themselves after developing symptoms (I → SQ). Pre-symptomatic or asymptomatic individuals could be isolated after receiving positive test results (I → Q). We assumed that 30% of newly infected individuals would develop symptoms after the incubation period and go to hospitals. Infected patients would recover according to their viral kinetic parameters (I, Q, SQ → R). We assume the basic reproductive number to be 2.5, which measures the spread of an infectious disease (see Table S2 in “Supplementary Materials” for more description of parameters used in the epidemic transmission models).

### Testing-isolation protocols

In our simulation, screening schemes started if the infection rate reached the initial prevalence of screening. Then, multiple rounds of screening were implemented to tests all participants. When one round of screening ended, a new round of screening would be initiated the next day until no unidentified infected individuals were mixed in the population or reached the longest simulation time (365 days).

Four screening schemes were evaluated over epidemic dynamics, including (1) pooled PCR screening, (2) individual PCR screening, (3) antigen screening (every 3 days), and (4) antigen screening (every 14 days). The best-in-class PCR assays demonstrate the limit of detection (LOD) of ~ 2 log10 viral RNA copies/ml, while LODs of currently approved assays range 2–3 log10 RNA copies/ml^51^. In population screening, LOD of PCR tests was set as 3 log10 viral RNA copies/ml, while antigen tests were less sensitive with a LOD of 5 log10 viral RNA copies/ml^48^. Although the specificities of RT-PCR tests^52^ and rapid antigen tests are close to 100%, false-positive results are impossible to avoid due to contamination during testing operations. Moreover, studies show that specificities of rapid antigen tests range from 98.4 to 100%, taking RT-PCR test results as reference^52–56^. We assumed individual PCR tests had a specificity of 99% and antigen tests had a specificity of 98% in this research. If test specificity in population screening can be improved to 100%, there will be no false-positive results.

Moreover, the delays in reporting lead to untimely isolation of infected patients, and they may transmit the disease before receiving the test results. The virus spread during turnaround time is also considered in the epidemic model. Individual PCR tests had a 1-day turnaround time, while pooling PCR tests had a 2-day turnaround time because it would take extra time to pool samples, conduct the second stage individual testing for positive pools, and report results. Antigen tests are point-of-care tests that offer results within 15–30 min, so they have no turnaround time.

For pooled and individual PCR screening, the main bottleneck was the limited laboratory testing equipment, which was difficult to obtain in a short time. We assumed that the PCR testing facilities could individually test the entire population in 60 days, so the basic daily test capacity was $\frac{N}{60}$ tubes. By pooling, we could screen more individuals using PCR tests. We also assumed laboratories would adapt the optimal pool size and sampling plans weekly to ensure full use of PCR testing capacity. In our simulation, the prevalence was known fully. In practice, pooled testing can accurately estimate prevalence across a broad range, from 0.02 to 20%^10^.

Samples in the pooled PCR test went through two stages. In the first stage, samples were collected and mixed into pools. One PCR test was conducted on each pool, and samples in positive pools advanced to the next stage, while samples in negative pools were labeled as negative. In the second stage, we performed individual PCR tests on positive samples from the first stage. A sample was labeled as positive only if the results in both stages were positive.

Antigen tests could be quickly scaled in large quantities. We assumed a high testing capacity of $\frac{N}{3}$ antigen kits per day (i.e., antigen testing can test all populations every 3 days) and a low testing capacity of $\frac{N}{14}$ antigen kits per day (i.e., antigen testing can test all populations every 14 days).

Both PCR and antigen tests generate false-negative and false-positive results, which were critical in population screening. In our simulation, we kept tracking viral load trajectories for each infected individual. A false-negative result would be generated if the viral load in pooled or individual samples was less than LOD. With a fixed specificity, false-positive results were randomly generated from pooled samples and individual samples with no virus.

Not everyone would like to be tested or isolated after receiving a positive result. Therefore, we assumed that 90% of the population were willing to participate in screening and follow the testing-isolation protocol. Participants without symptoms went back to their normal life after receiving negative results, despite a possibility of false-negative results. All participants who were not isolated would participate in the next round of screening.

In population screening, an overwhelming number of false-positive results might cause unnecessary isolation of healthy people and reduce their compliance. To avoid this, we assumed that participants who got positive results would be confirmed by another testing method (usually a different testing technology) to exclude false-positive results. Only confirmed cases would be isolated until recovery. We record the number of false-positive results to represent the number of confirmatory tests.

### Cost analysis

Using simulation results, we evaluated the cost of all screening schemes under COVID-19 pandemics. The cost of PCR tests can be categorized into two parts: (1) reagents/consumables, like RNA extraction kits, and (2) labor costs, like pooling and reporting labor.

The cost of employing technicians to do RT-PCR tests was $36.50 per h^17^. Antigen screening only costs $10 per testing kits^30^. All cost data come from the US data. The categorized costs are allocated per test or per sample, according to Abdalhamid et al.^17^, as shown in Table S3. Then we can evaluate the cost of four screening schemes based on simulation results and categorized costs. We assume the unit costs of materials and labor remain constant throughout the simulation.

## Results

### Impacts of viral load distribution on test sensitivity without testing-isolation

For each infected individual, the probability of detecting an infection (i.e., sensitivity) varies as their individual-level viral load changes during the infection. Sample type also affects the test sensitivity, according to a recent review for systematic meta-analysis of the sensitivities of PCR tests using different sample types^57^. We generated individual-level viral load trajectories based on Cleary et al.^10^, which derived viral load kinetics using pharyngeal swabs^50^. This type of sample is the most used in population screening. We did not distinguish the viral load trajectories of symptomatic and asymptomatic individuals for the following reasons: first, the newest study revealed that viral loads are similar between asymptomatic and symptomatic individuals but different in viral clearance time^58^. Then, our study focuses on asymptomatic and pre-symptomatic individuals who have similar viral load trajectories before developing symptoms. The symptomatic individuals do not participate in population screening because they need clinical diagnostic tests.

**Figure 2:**
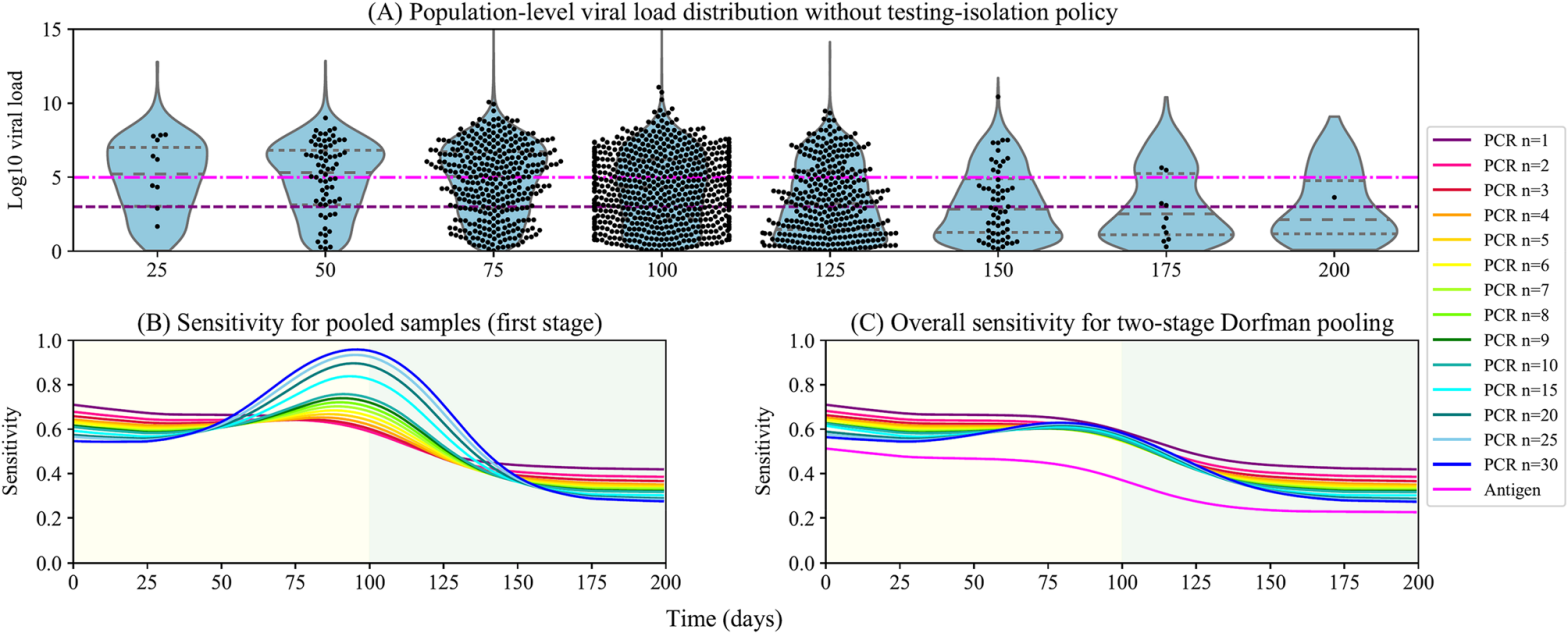
Viral load distribution of infected individuals and regressed sensitivities throughout the outbreak without testing-isolation. (**A**) Viral load distribution of infected individuals without testing-isolation policy. At each point, black dots are viral loads of infected individuals in a random sample of 3000 individuals. The grey dashed lines in the violin graph (from bottom to top) are the first, second, and third quartiles of viral load distribution of infected individuals. The limit of detection of PCR (purple dotted line) and antigen tests (pink dash-dotted line) are 3 and 5 log10 viral RNA copies/ml, respectively. (**B**) Sensitivity of PCR pooled samples, individual PCR tests, and antigen tests. ‘PCR n = 1’ is the individual PCR test, while ‘PCR n = k’ represents a pool size of k. 'Antigen' is the sensitivity for individual antigen tests. (**C**) The overall sensitivity of Dorfman pooling. Sensitivities of individual PCR tests (solid purple line) and antigen tests (solid pink line) are also included. The yellow area represents the rising phase of the epidemic (Day 0–100), and the green area represents the falling phase (Day 100–200).

For population screening, the sensitivities are influenced by the population-level viral load distributions, which change as the epidemic evolves^10,30^. As is shown in Fig. 2A, the population-level viral load distribution evolves throughout the epidemic outbreak. We refer interested readers to see Hay et al. for more details about the cross-sectional population-level viral load distribution^30^.

Sensitivities of pooled testing are more complicated than individual testing. Due to the dilution effects, sensitivities of pooled testing vary with pool sizes and population-level viral load distribution. There are three sensitivities of interest: the sensitivity of PCR tests using the individual sample, ${S}_{ei}(t)$, the sensitivity of PCR tests using pooled samples, ${S}_{ep}(t,n)$, and the overall sensitivity of two-stage pooled testing, ${S}_{ed}(t,n)$.

First of all, we simulated an epidemic outbreak without testing-isolation policy. The outbreak peaked at day 100 (Fig. 2A). The population-level viral load distribution was simulated by generating the viral load of each infection during the outbreak. Then we regressed the sensitivities of pooled PCR tests, individual PCR tests, and antigen tests with the time since the beginning of the pandemic and the pool size. To reduce the stochastic error of these sensitivity estimations, we ran the simulation 8 times and used LOWESS regression to fit ${S}_{ei}(t)$, ${S}_{ep}(t,n)$, and ${S}_{ed}(t,n)$ numerically, as is shown in Fig. 2B,C.

In practice, the large-scale population screening could change the population-level viral load distribution by isolating identified infected individuals. We will discuss the impact of testing-isolation in the next section.

As shown in Fig. 2A, the population-level viral load distributions have a big difference in the epidemic growth stage (Day 0–100) and decline stage (Day 100–200). During the epidemic growth, a fair percentage of infected individuals are in the early stage of infection, and their viral loads are close to peak viral load, while most people gradually recover with waning viral loads during the epidemic decline. The median, first, and third quartiles of population-level viral load distribution decreases during the pandemic as more infected individuals enter the recovery phase of the disease. Most people will have undetectable viral loads after 200 days.

Overall, the sensitivities of Dorfman pooling PCR, individual PCR, and antigen tests have downward trends during the outbreak (Fig. 2C) due to the falling population-level viral load distribution (Fig. 2A). However, the sensitivities of pooled samples (the first stage of Dorfman pooling) fluctuate drastically, showing the enhancement effect at a high prevalence and the dilution effect at a low prevalence (Fig. 2B). When the prevalence is very high, almost all pools return positive results, and false negatives with low viral load can be 'rescued' by pooling with high viral load samples, i.e., the enhancement effect. On the other hand, when the prevalence is low, positive samples are diluted with more negative samples, and the average viral load decreases, leading to possible loss of sensitivity, i.e., the dilution effect.

Due to the systematic error of hierarchical operation, the overall sensitivities of two-stage Dorfman pooling cannot surpass individual PCR testing (Fig. 2C). Moreover, the hierarchical procedures also take longer to report results, which weakens the effect of testing-isolation policies. Thus, the combinatorial pool design, where samples are split into multiple non-hierarchical pools, was proposed to facilitate reporting results and efficiency (see Clearly et al. for more details^10^). However, as combinatorial pooling is complicated in splitting samples into several pools, Dorfman pooling has the advantage of simplicity in population screening.

As expected, the sensitivity of individual PCR tests is higher throughout the pandemic than pooled PCR and antigen tests. Although the pooled PCR tests lost some sensitivity due to the systematic error of hierarchical operation and dilution effects, they still have higher sensitivity than antigen tests. In our model, the mean sensitivity of individual PCR testing is 67% during epidemic growth, 46% during the epidemic decline, and 56% across the whole epidemic. A recent study estimated the overall sensitivity of home self-swabbing RT-PCR based on a single swab as 79% (77%, 81%)^59^. The difference from the literature is due to our higher LOD for PCR tests in population screening, so the test sensitivity is lower. Taking the maximum pool size 30 as an example, the overall sensitivity of Dorfman pooling is 59% during epidemic growth, 36% during the epidemic decline, and 48% across the whole epidemic.

Moreover, the mean sensitivity of antigen tests is 47% during epidemic growth, 25% during the epidemic decline, and 40% across the whole epidemic. Comparing with PCR test results, studies found that the antigen test sensitivity was 41.2% for asymptomatic participants and 80.0% symptomatic participants^60^. As the positive results in PCR tests do not represent all “true positive results”, studies estimated the sensitivity of antigen tests as 48.6% under the assumption of a 10% false-negative rate of PCR tests^53^.

### Impacts of testing-isolation on viral load distribution and sensitivities

**Figure 3:**
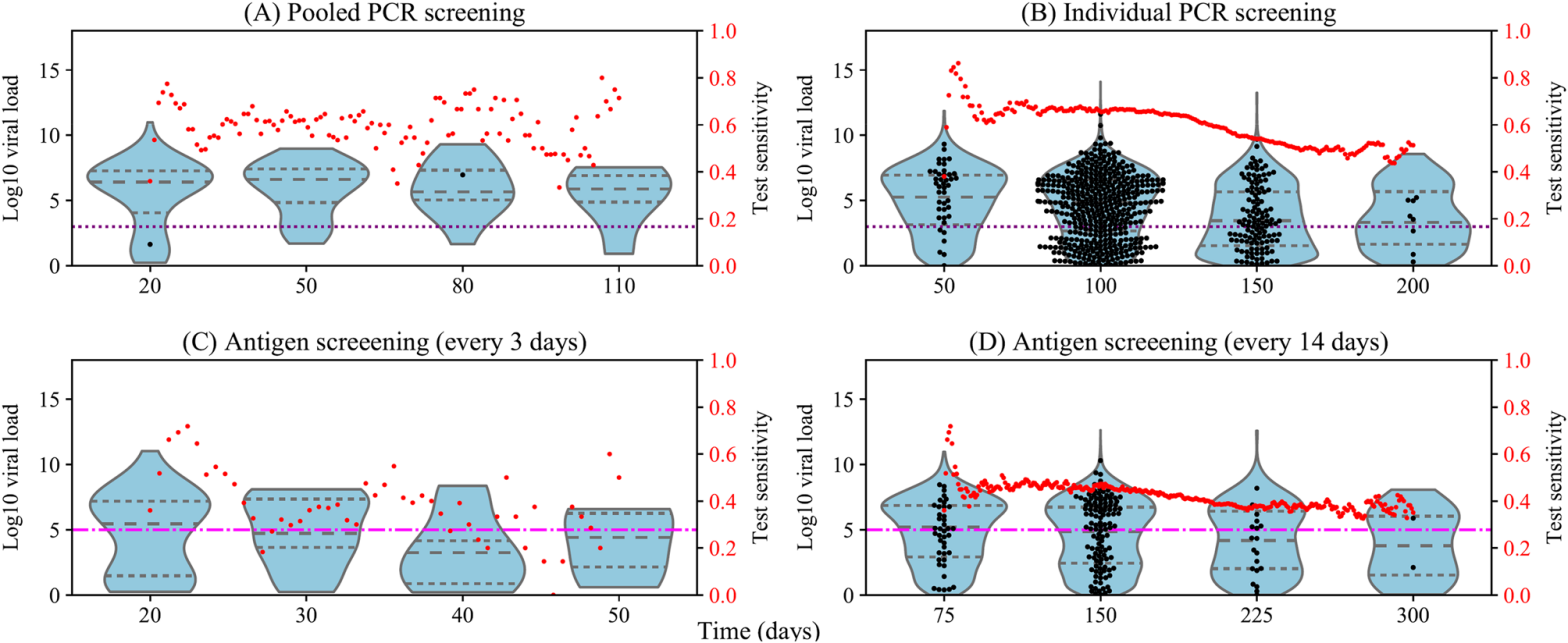
Viral load distribution of infected individuals and test sensitivity under different screening schemes with testing-isolation (initial prevalence of screening 0.1%). The grey dashed lines in the violin figure (from the bottom to the top) are the first, second, and third quartiles of viral load distribution of unidentified infected individuals in 3000 randomly sampled individuals. The black dots are viral loads of sampled infected individuals. The red dots represent the real-time sensitivity of screening which changes with the population-level viral load distribution. Viral load distributions of unidentified infected individuals and test sensitivities are under (**A**) pooled PCR screening, (**B**) individual PCR screening, (**C**) antigen screening (every 3 days), and (**D**) antigen screening (every 14 days). The limit of detection of PCR (purple dotted line) and antigen tests (pink dash-dotted line) are 3 and 5 log10 viral RNA copies/ml, respectively. The horizontal axes vary due to the different end times of outbreaks.

In population screening, the testing-isolation process will affect the population-level viral load distribution, influencing the test sensitivities. Therefore, we incorporated the testing-isolation policy into the epidemic transmission model and weekly re-calculated all sensitivity parameters and the optimal pool size by simulating random pooled testing of all pool sizes. Figure 3 shows the viral load distribution of unidentified infected individuals and test sensitivity under different screening schemes. Population screening starts when prevalence reaches 0.1% and ends when all infected individuals are isolated or when the running time reaches the maximum. The horizontal axes vary due to the different end times of population screening. Moreover, the optimal pool size of pooled PCR screening maintains 30 throughout the outbreak (Fig. 5A) because of the low prevalence.

When the testing-isolation process could remove most infected individuals from the transmission chain, test sensitivity does not drop substantially during the epidemic decline (Fig. 3A,C). As shown in Fig. 2C, without the testing-isolation process, overall sensitivities of pooled PCR, individual PCR, and antigen tests fall off during epidemic decline. In contrast, the sensitivity under pooled PCR screening does not decrease during the epidemic decline because of the high population-level viral load distribution throughout the outbreak (Fig. 3A). As is shown in Fig. 3A, the first, second, and third quartiles of viral load distribution of unidentified infected individuals under pooled PCR screening are higher than the LOD of PCR tests throughout the outbreak. As individual PCR screening and antigen screening (every 14 days) cannot remove most infected individuals from the transmission chain, the sensitivity drop obviously during epidemic decline (Fig. 3B,D).

### Impacts of population screening on epidemic dynamics

**Figure 4:**
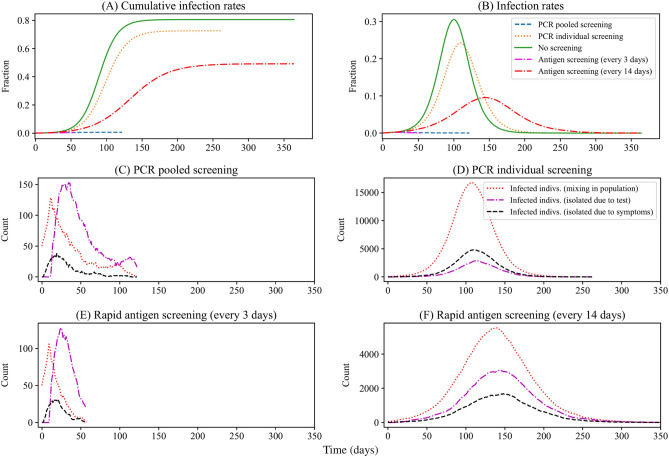
Epidemic dynamics under different screening schemes with testing-isolation (initial prevalence of screening 0.1%). (**A**) Cumulative infection rates $\left( {1 - \frac{S}{N}} \right)$ and (**B**) infection rates $\left( {\frac{I + Q + SQ}{N}} \right)$ of different screening schemes over time. For each screening scheme, trajectories of infected individuals ($I,Q,SQ$) under (**C**) PCR pooled screening, (**D**) PCR individual screening, (**E**) antigen screening (every 3 days), and (**F**) antigen screening (every 14 days) were plotted separately. Note the variation in the vertical axes.

We evaluated different population screening schemes under a dynamic transmission model described in our methods. As shown in Fig. 4A,B, without screening, the susceptible population was quickly infected through contact with pre-symptomatic or asymptomatic individuals. Pre-asymptomatic individuals were isolated after developing symptoms, while asymptomatic individuals continued to spread the virus without knowing their infection. The epidemic outbreak would decline as a large population gained immunity.

**Figure 5:**
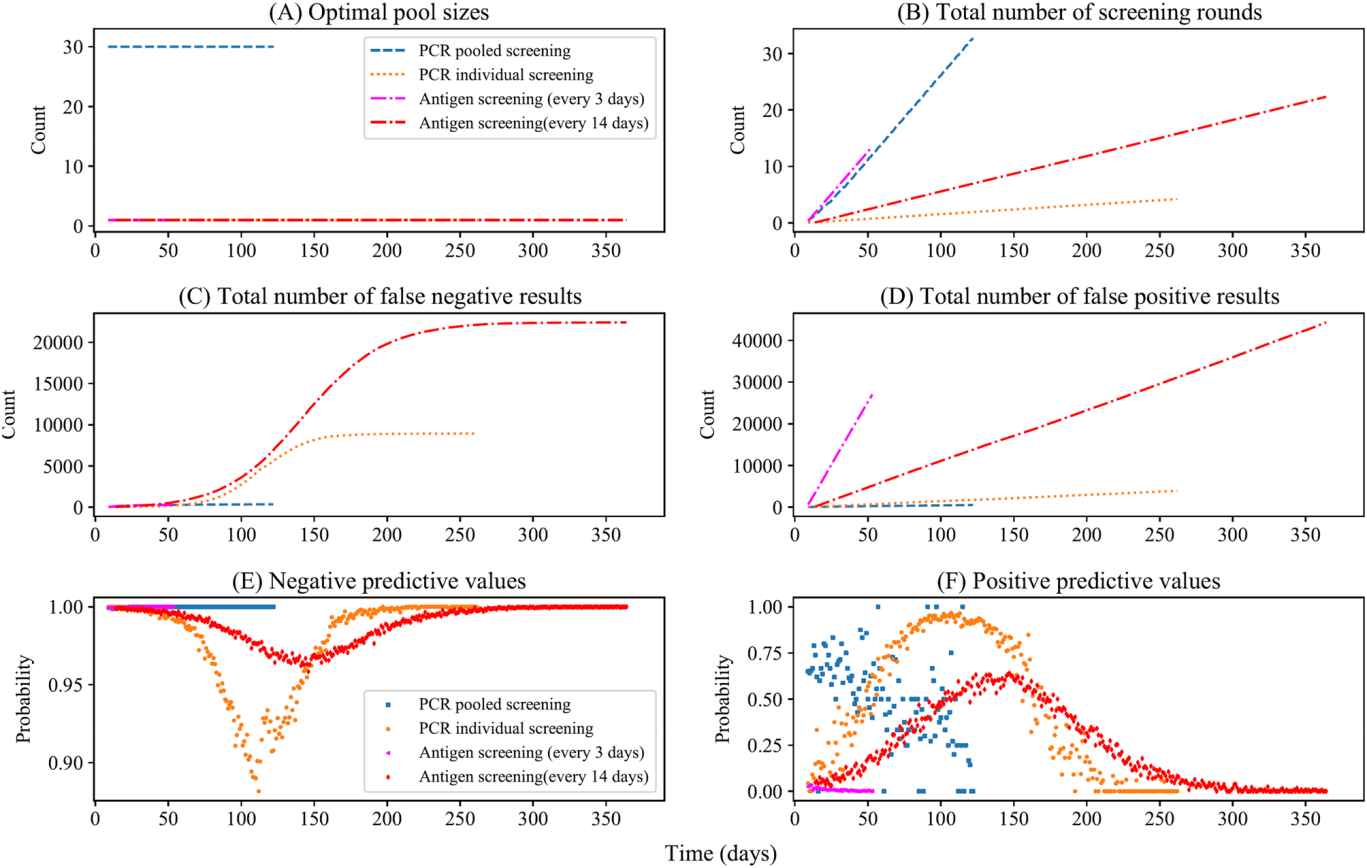
Optimal pool size and test results under different screening schemes with testing-isolation (initial prevalence of screening 0.1%). (**A**) Optimal pool sizes for pooled PCR screening stayed at the maximum value because of low prevalence. The pool size of individual PCR and antigen are all one sample. (**B**) The total number of screening rounds. Pooled PCR screening has a similar testing frequency with antigen screening (every 3 days), while antigen screening (every 3 days) ends the virus spread earlier than pooled PCR screening. (**C**) The total number of false-negative results. Pooled PCR screening and antigen screening (every 3 days) have few false-negative results, while antigen screening (every 14 days) has the largest number of false-negative results. (**D**) The total number of false-positive results. Pooled PCR screening has the fewest false-positive results, while antigen screening has a large number of false-positive results. (**E**) Negative predictive values. The negative predictive values of pooled PCR screening and antigen screening (every 3 days) are higher than those of individual PCR screening and antigen screening (every 14 days) at most points during the pandemic, except the initial periods. (**F**) Positive predictive values. Pooled PCR screening has the highest positive predictive values at most points during the pandemic. Note the variation in the vertical axes.

Pooled PCR screening or antigen screening (every 3 days) could reverse the outbreak**.** As is shown in Fig. 4C,E, compared to no screening, population screening using pooled PCR tests or antigen tests (every 3 days) could immediately reverse the outbreak, as most unidentified infected individuals were isolated after testing. In addition, although the daily number of people taking tests was similar for the pooled PCR screening and antigen screening (every 3 days) (Fig. 5B), antigen screening (every 3 days) could reverse and end the epidemic spread earlier. It resulted in fewer total infections than pooled PCR screening because the 2-day turnaround time of pooled testing delayed the isolation of infected individuals and diminished the effectiveness of pooled screening, offsetting some sensitivity advantages of PCR tests.

Antigen screening (every 14 days) could mitigate the outbreak. Although antigen tests are less sensitive than PCR tests, antigen screening (every 14 days) could isolate a large number of infected individuals (Fig. 4F) and contain the epidemic to a low prevalence (Fig. 4B), winning time for the development of vaccines and treatments.

Individual PCR testing was ill-suited for population screening owing to its limited effects on isolating infected individuals (Fig. 4D). As is shown in Fig. 4A,B, individual PCR tests only partially mitigated the total infection and flattened the curve because of limited testing capability. The epidemic faded as people developed herd immunity after recovering from COVID-19. The pattern was similar to that of the no-screening scenario.

### Accuracy of different screening schemes during the pandemic

The accuracy of population screening was evaluated from two aspects: the number of testing errors and the predictive value of test results. The number of testing errors has a direct impact on epidemic dynamics and screening operations. The predictive value of test results helps to interpret the test results.

False-negative results are more consequential, as infected individuals with false-negative results spread the virus unwittingly. The impacts of false-negative results could be rescued by multiple rounds of screening, i.e., the repeated screening. As is shown in Fig. 5C, pooled PCR screening and antigen screening (every 3 days) had fewer false-negative results than individual PCR screening and antigen screening (every 14 days) in the long run. At the beginning of the screening, pooled PCR and antigen screening generated false-negative results at higher rates than individual screening because of low sensitivity and high testing throughput. However, as prevalence decreased, thanks to pooled PCR screening or antigen screening (every 3 days), the cumulative number of false-negative results remained at a low level. By contrast, the individual PCR screening and antigen screening (every 14 days) accumulated a large number of false-negative results (Fig. 5C) as cumulative infections increased (Fig. 4A).

In population screening, false-positive results are also problematic, as people with false-positive results need another confirmatory test or isolation. Moreover, false-positive results may discourage people's compliance with the testing-isolation policy. Pooled PCR screening had the fewest false-positive results, followed by individual PCR screening, antigen screening (every 14 days), and antigen screening (every 3 days). As the specificity assumed a fixed value, the number of false-positive results was proportional to the number of total tested individuals and increased almost linearly over time. Although pooled PCR screening tested the largest number of people, it generated the fewest false-positive results (Fig. 5D) by testing each positive sample twice in the Dorfman pooling operations. The hierarchical tests ruled out some false-positive results in Dorfman pooling.

Positive predictive value (PPV, the probability that subjects with a positive result truly have the disease) and negative predictive value (NPV, the probability that subjects with a negative result truly do not have the disease) help interpret testing results. PPVs and NPVs incorporate the information on prevalence, pool size, test sensitivity, specificity, and testing procedures. Thus, high PPVs and NPVs correspond to reliable positive and negative results, respectively.

According to our simulation, pooled PCR and antigen testing (every 3 days) could increase their NPVs by suppressing the prevalence, as NPVs increase as prevalence decreases. As is shown in Fig. 5E, in our simulation, pooled PCR and antigen screening (every 3 days) had high NPVs during the pandemic, despite their low sensitivities.

In our simulation, PCR screening had high PPVs during the pandemic, especially the pooled PCR screening, as shown in Fig. 5F. Pooled PCR testing increases PPVs by repeated testing on positive samples in two-stage operations. Thus, pooled PCR tests had a much higher PPV than antigen tests and individual PCR tests under very low prevalence. Although the specificity of antigen (98%) tests was slightly lower than that of PCR tests (99%), antigen screening had much lower PPVs than PCR screening, especially at low prevalence.

### Cost-effectiveness of different screening schemes during the pandemic

**Figure 6:**
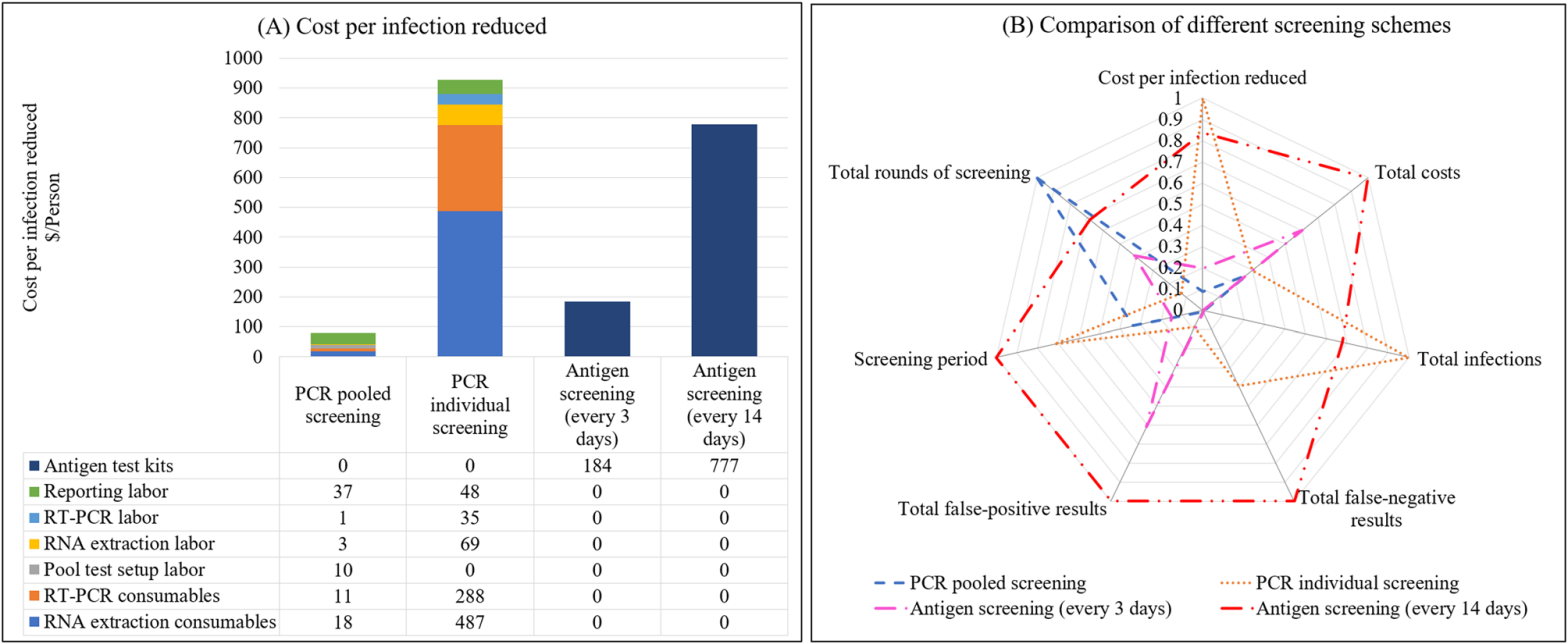
Comparison of different screening schemes (**A**) Cost per infection reduced. To end the virus spread, pooled PCR screening has the least total cost (no more than $100 per infection reduced), within which labor cost accounts for more than half of the total cost. Antigen screening (every 3 days) costs more than $150 per infection reduced. The cost of antigen screening comes from antigen test kits. Although individual PCR screening has a high sensitivity, it is most costly to identify one infected patient due to the limited capacity. (**B**) Comparison of different screening schemes. Four testing schemes were compared from seven aspects, including cost per infection reduced, total cost, total infections, the total number of false-negative results, total number of false-positive results, total rounds of screening, and the duration of screening. All data in (**B**) are normalized into the range of 0 and 1.

From the cost-effectiveness aspect, pooled PCR screening had the lowest cost to reduce one infection, while individual PCR screening was most costly but had the highest total infections, as shown in Fig. 6A. Moreover, pooling and reporting labor costs took more than half of the total cost for pooled PCR screening.

Although the antigen test kit can be used for self-testing and is nearly half the price of individual PCR tests, it requires a high testing frequency (e.g., testing every 3 days) to suppress the pandemic, leading to a high total cost and a large number of false-positive results (Fig. 6B). In our simulation, if the cost of antigen test kits fell by 60%, it would be cheaper to use antigen screening (every 3 days) than pooled PCR screening to identify one infected individual. In addition, as shown in Fig. 4B, antigen screening (every 3 days) could stop the pandemic in the shortest time, which is critical for economic recovery. Although individual PCR screening has the highest sensitivity, it is most costly to identify one infected patient due to the low efficiency.

In practice, pooled screening is a labor-intensive process that poses a significant challenge to both the healthcare system and participants, as healthcare workers need to collect samples from people more than 30 times in 100 days, while antigen testing is more convenient for participants and reduces the burden on laboratories. However, antigen screening generates more false-positive results, leading to unnecessary isolation or confirmatory tests. On the other hand, pooled PCR screening generates the fewest false-positive results because of the two-stage operation on positive samples.

### Impacts of the initial prevalence of population screening

The initial prevalence for implementing population screening is critical for reversing the outbreak, especially for pooled PCR screening. If the initial prevalence was lower than 0.1%, the pooled PCR screening could quickly reverse the growth of the outbreak (Fig. 4B). Pooled PCR screening entered an accelerating process after the pandemic was under control. As is shown in Fig. 5B, the first round of pooled screening took 8 days, with a 2-day delay in reporting. After several rounds of screening, pooled PCR screening could test all the participants within 3–4 days, which was similar to the antigen screening (every 3 days).

**Figure 7:**
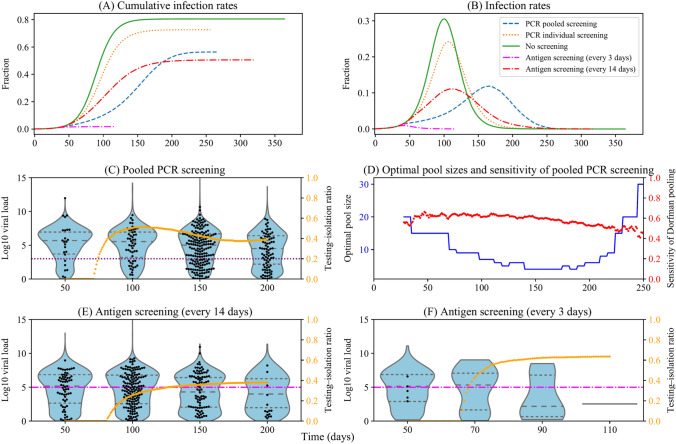
Epidemic dynamics, viral load distributions, and testing-isolation ratios under difference screening schemes with testing-isolation (initial prevalence of screening 0.5%). (**A**) Cumulative infection rates $\left( {1 - \frac{S}{N}} \right)$ and (**B**) infection rates $\left( {\frac{I + Q + SQ}{N}} \right)$ of different screening schemes over time. Viral load distributions and the testing-isolation ratios are under (**C**) PCR pooled screening, (**E**) antigen screening (every 14 days), and (**F**) antigen screening (every 3 days). The grey dashed lines in the violin figure (from the bottom to the top) are the first, second, and third quartiles of viral load distribution of infected individuals in 3000 randomly sampled individuals. The black dots are viral loads of sampled infected individuals. The orange dots represent the testing-isolation ratio, the proportion of isolated individuals due to tests to the cumulative infected individuals. (**D**) The optimal pool sizes (blue line) and the corresponding sensitivities of the Dorfman pool (red dots). The limit of detection of PCR (purple dotted line) and antigen tests (pink dash-dotted line) are 3 and 5 log10 viral RNA copies/ml, respectively. Note the variation in the horizontal and vertical axes.

Pooled PCR screening needs to be implemented at a low initial prevalence to reverse the pandemic, as the effects of pooling PCR screening become weaker as prevalence increases. When the initial prevalence is 0.1%, pooled PCR screening can reverse the outbreak, similarly to the antigen screening (every 3 days). However, if the initial prevalence reaches 0.5%, pooled PCR screening could only mitigate the outbreak rather than reversing the growth of an outbreak (Fig. 7A,B). During the early stage of population screening, pooled PCR screening (Fig. 7C) isolated a higher ratio of infected individuals (i.e., testing-isolation ratio) than antigen screening every 14 days (Fig. 7E). As prevalence increases, the optimal pool size decreases (Fig. 7D), and the testing-isolation ratio of pooled PCR screening gets worse antigen screening every 14 days (Fig. 7C,E).

Increasing antigen screening frequency could reverse the outbreak when the initial prevalence is high (e.g., 0.5%). For example, antigen screening every 3 days could isolate more than 60% of infected individuals (Fig. 7F). We assume that 30% of infected individuals will go to hospitals after developing symptoms. Most infected individuals will be isolated under antigen screening (every 3 days).

**Figure 8:**
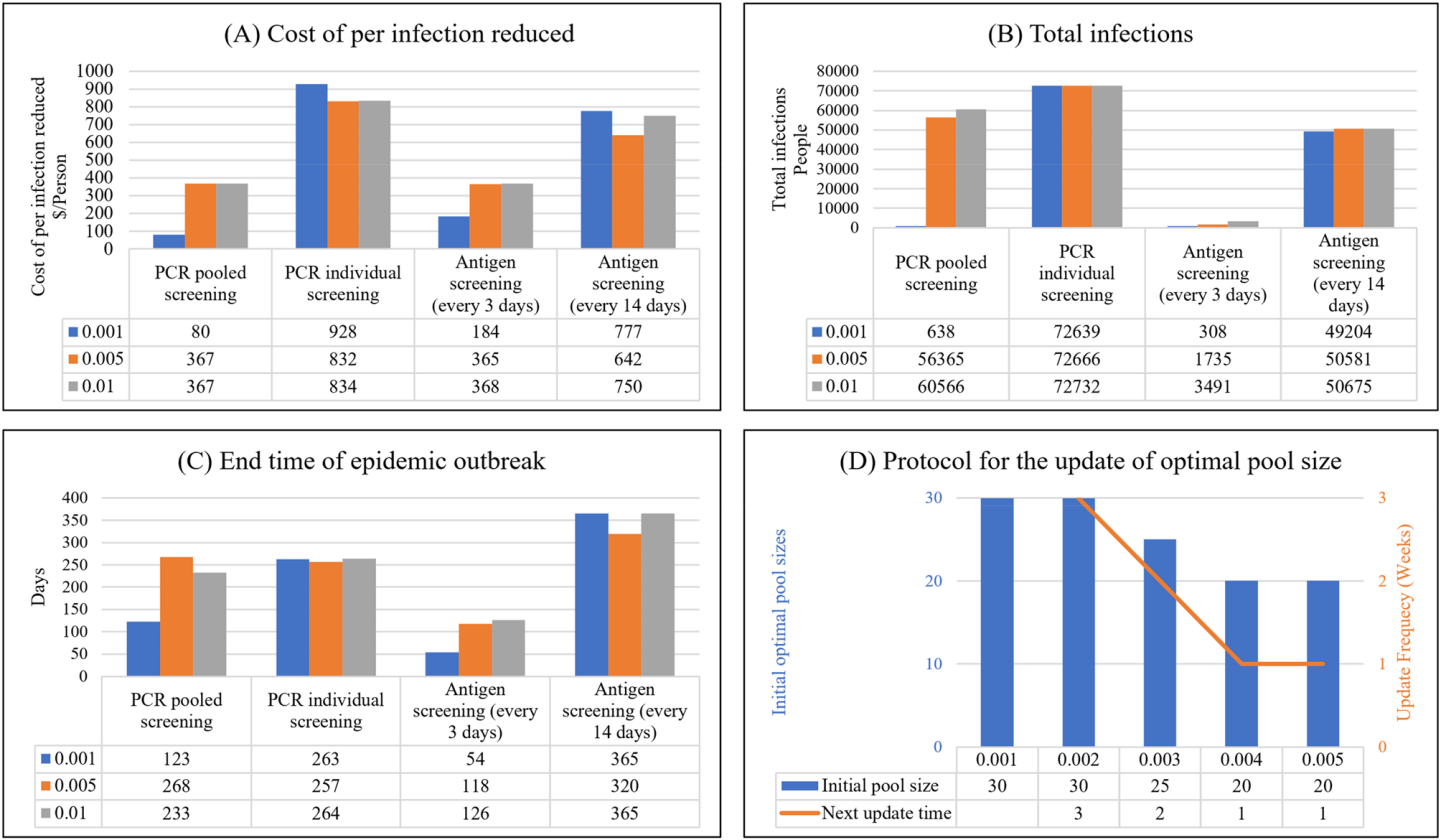
Comparison of different screening schemes with different initial prevalence. When screening starts at prevalence rates of 0.1%, 0.5%, and 1%, (**A**) pooled PCR screening takes the least cost to reduce one infection, followed by antigen screening (every 3 days), antigen screening (every 14 days), and individual PCR screening; (**B**) antigen screening (every 3 days) has the fewest total infections, while individual PCR screening has the largest infected population. (**C**) The end time of the epidemic outbreak. Antigen screening (every 3 days) will end the ep idemic earlier than PCR pooled screening. Antigen screening (every 14 days) will not end the pandemic within 1 year. Note that our simulation ends after 365 days. Individual pooled testing ends the epidemic earlier than antigen screening (every 14 days) by developing herd immunity. (**D**) The protocol to update the optimal pool size. When the preval ence i s lower than 0.1%, the optimal pool size is 30 and does not need to be changed. As prevalence exceeds 0.2%, the optimal pool size is 30 and needs to be changed after 3 weeks. As prev alence exceeds 0.3%, the optimal pool size is 25 and needs to be changed after 2 weeks. As prevalence exceeds 0.4%, the optimal pool size is 20 and needs to be changed after 1 week. As prevalence exceeds 0.5%, optimal pool size should be updated weekly according to Fig. 7D.

We further compared different testing strategies and technologies by simulating population screening starting at different prevalence rates, e.g., 0.1%, 0.5%, and 1%. When prevalence was low, pooled PCR screening was the most cost-effective scheme (Fig. 8A); antigen screening (every 3 days) had the fewest total infections (Fig. 8B) and ended the outbreak at the earliest (Fig. 8C); while individual PCR tests were not suitable for population screening (Fig. 8A,B).

However, the effectiveness of pooled PCR screening diminished as the initial prevalence of screening increased, as shown in Fig. 8B,C, because the optimal pool size was sensitive to the prevalence. Thus, when pooled PCR screening started at a higher prevalence, pooled screening could not reverse the epidemic. Therefore, pooled PCR screening should be implemented as early as possible.

### A protocol to update optimal pool size

As it may not be feasible to adjust the pool size frequently, we propose a simple protocol to determine the optimal pool size and the next update time at different prevalence rates, as shown in Fig. 8D. When the prevalence remains lower than 0.1%, the optimal pool size is 30 and requires no update. If the prevalence exceeds 0.2%, the optimal pool size remains 30 but needs to be updated after 3 weeks. If the prevalence exceeds 0.3%, the optimal pool size decreases to 25 and needs to be changed after 2 weeks. If the prevalence exceeds 0.4%, the optimal pool size is 20 and needs to be changed after 1 week. Finally, as prevalence exceeds 0.5%, optimal pool size should be updated weekly.

## Conclusion and discussion

Our results show that the testing-isolation policy changes the population-level viral load distributions, influencing the test sensitivities. When the testing-isolation process could remove most infected individuals from the transmission chain, the population-level viral load distribution throughout the outbreak remains high, and test sensitivity does not drop substantially during the epidemic decline.

Our results indicate that high-frequency population screening at low prevalence enabled by pooled PCR and antigen tests is a cost-effective method to reduce total infections and potentially end the viral transmission of SARS-CoV-2. Although individual PCR tests are useful in clinical diagnosis because of their high accuracy, they are ill-suited for population screening owing to their limited capacity.

Although pooled PCR testing sacrifices accuracy for efficiency and has a long turnaround time for reporting results, pooled PCR screening remains a cost-effective method to contain the epidemic or even stop the virus transmission, especially at the low prevalence. Compared to individual PCR and antigen testing (every 14 days), pooled testing can test more people with existing equipment and isolate more pre-symptomatic and asymptomatic infected patients to prevent future infections. Moreover, pooled testing generates much fewer false-positive results because of the two-stage testing procedure on positive samples. In population screening, low sensitivity can be rescued by increasing testing frequency, while false-positive results lead to unnecessary confirmatory tests or isolation. Our results were consistent with previous research that repeated testing could effectively contain the epidemic^48,49^.

For developing countries, building up the pooled PCR warning and screening systems could expedite responses to the possible epidemic outbreaks in the future. First of all, PCR testing works for possible virus variations and other infectious diseases. Then, the labor cost, which takes more than half of the total pooled PCR screening cost, is much cheaper in developing countries. Besides, the epidemic warning systems could detect the outbreak at an early stage. The earlier pooled PCR screening is implemented, the earlier the epidemic outbreak will end, reducing total infections and costs. As the prevalence increases, pooled tests will use a smaller pool size, and the test capacity expansion effect will diminish. Thus, implementing pooled PCR testing at high prevalence cannot reverse the direction of the epidemic outbreak, while high-frequency antigen testing could reverse the epidemic at a high cost. However, the development of antigen tests is difficult for many developing countries because it requires many scientists and lots of funding. In addition, importing antigen test kits is expensive, and the supply has great uncertainty. Therefore, we suggest developing countries build up pooled PCR warning and screening systems to suppress the outbreaks as early as possible for the above reasons.

Our results indicate that the pooled PCR can be more cost-effective than high-frequency antigen testing. However, if manufacturers can significantly reduce the price of antigen testing kits and massively raise production to meet the high testing frequency, high-frequency antigen testing would be better for population screening than pooled PCR screening. Nevertheless, high-frequency antigen screening still has problems even if the price is further reduced. Due to the low specificity, false-positive results are typical when antigen tests are used in a population with a low prevalence. Therefore, positive results of antigen tests for participants without symptoms should be treated as presumptively positive results until confirmed by another test as soon as possible^51^. Otherwise, a high percentage of the population will be wrongly isolated, which may cause social panic and low compliance of participants. Thus, healthcare providers should offer more information on interpreting test results and organize timely follow-up testing to rule out false-positive results during antigen screening.

In addition, workflows of pooled PCR screening can be modified to improve cost-effectiveness further. (1) Pooling samples immediately upon collection can save pooling labor and consumables, especially when the prevalence is very low. For example, in China, ten samples are collected in one tube as a pool for asymptomatic population screening. If the pool tests positive, all individuals will be individually resampled and re-tested. (2) It is beneficial to advise participants to self-quarantine to prevent possible onward transmission until receiving negative test results. This is equivalent to reducing the turnaround time, as a delay in reporting results can significantly diminish the effectiveness of screening^48^. (3) Household-pooled testing saves resources and labor by constructing pools out of individuals that belong to the same household. For countries with limited testing capacity, if the pool tests positive, all family members will be isolated because they are close contacts to each other^8^. (4) Application of highly inter-related digital technologies can also improve the effectiveness of population screening, the response to tests results, and the compliance of populations^39–41^. These technologies include the Internet of Things^42,43^, biosensors^44,45^, health information technology^46^, telemedicine^47^, and machine learning algorithms^44,47^.

Our analysis was subject to several limitations. First, although we incorporated testing in a stochastic epidemic model with individual-level trajectories, we did not incorporate the population mobility, contact patterns, age, or gender differences, considered in other agent-based models. Second, our model can be extended to a more detailed transmission model and re-parameterized to evaluate different testing schemes once more detailed data are available. Moreover, our model can be further extended to incorporate large-scale contact tracing to improve the cost-effectiveness of population screening.

## Supplementary Information

Supplementary Information.

## Data Availability

All code is available for free use at: https://github.com/JL-YU/Flexible-Testing.
