# Supplementary material for: Optimizing and evaluating PCR-based pooled screening during COVID-19 pandemics
Source: Sci Rep. 2021 Nov 2;11:21460. doi: 10.1038/s41598-021-01065-0 (PMC8564549; doi:10.1038/s41598-021-01065-0)
Supplement: Supplementary file 1 — Supplementary Information. [file 41598_2021_1065_MOESM1_ESM.docx]

**Appendix**

**Virus kinetics**

We adopted the viral load kinetic model proposed by Cleary et al. which simulated the population-level viral load distribution along the epidemic curve by generating individual-level viral load trajectories based on empirically virus kinetics^10^. The log10 viral load at time $t$ of infected individual $i$ be $v^{i}\left( t \right)$ is given by:

$$v^{i}\left( t \right)=\left\{ \begin{aligned} 0, &t_{inf}^{i}<t\leq t_{inf}^{i}+t_{g}^{i} \\ \frac{\alpha_{i}}{t_{p,i}}\left( t-t_{inf}^{i} \right), &t_{inf}^{i}+t_{g}^{i}<t\leq t_{inf}^{i}+t_{g}^{i}+t_{p}^{i} \\ \alpha_{i}-\frac{\alpha_{i}}{t_{inc}^{i}+t_{w}^{i}-\left( t_{g}^{i}+t_{p}^{i} \right)}\left( t-\left( t_{inf}^{i}+t_{g}^{i}+t_{p}^{i} \right) \right), &t_{inf}^{i}+t_{g}^{i}+t_{p}^{i}<t\leq t_{inf}^{i}+t_{inc}^{i}+t_{w}^{i} \\ 0, &t>t_{inf}^{i}+t_{inc}^{i}+t_{w}^{i} \end{aligned} \right.$$

The time of individual $i$ getting infected is $t_{inf}^{i}$. After infection, the viral load of infected individual has a latent period $t_{g}^{i}$ when viral load is 0. Then, the viral load of infected individual $i$ takes $t_{p}^{i}$ time to reach its peak value $\alpha_{i}$. Later, the viral load wanes after $t_{w}^{i}$ time. Although all individuals are assigned an incubation period $t_{inc}^{i}$, only symptomatic individuals will have symptom onset.

Based on the viral load trajectories of COVID-19 patients in real world, Cleary et estimated the posterior distributions of viral kinetic parameters (shown in Table S1) using Markov chain Monte Carlo methods, see Figure S11, S12 in their supplementary materials^9^.

**Table S1. Description of parameters used in the viral kinetics.**

| **Parameter** | **Description** | **mean** | **std** |
| --- | --- | --- | --- |
| $\alpha$ | Individual peak log 10 viral load | 8.007 (log10(copies/ml)) | 1.137 |
| $t_{p}$ | Individual time from viral occurrence to peak value | 1.501 (days) | 0.867 |
| $t_{inc}$ | Individual time from infection to symptom onset | 5.492 (days) | 2.346 |
| $t_{g}$ | Individual latent period | 1.485 (days) | 0.863 |
| $t_{w}$ | Individual time from peak viral load to undetectable | 15.192 (days) | 4.440 |

**Derivations of $\boldsymbol{CPR(p,n)}$.**

The expected number of tests required to identify an infected patient, $CPR(n)$, evaluates the overall performance of the efficiency and effectiveness of the testing strategy. Thus, we first derived the indicators of efficiency and effectiveness as functions of the prevalence $p$, pool size $n$, group sensitivity for pooled sample $S_{ep}$, sensitivity for individual sample $S_{ei}$, sensitivity for Dorfman pooled testing $S_{ed}$, and specificity $S_{p}$. At a given time $t$ days from the start of the pandemic. The expected number of tests for each subject $\mathbb{E[}T]$ measures the efficiency of different test strategies^37^. For individual testing, each subject only takes one test. For pooled testing of size $n$ $(n\geq2)$, the pooled sample is tested in the first stage. If the pooled sample tests negative, then all subjects in this pool are classified as negative. If pooled sample tests positive, then all the subjects in this pool need to be retested individually. Note that we should consider the false positive and false negative results in the first stage. If $n\geq2$, the per-subject expected number of tests $\mathbb{E[}T]$ is:

$\mathbb{E[}T]=\left( 1-p \right)^{n}\left( \frac{1}{n}S_{p}+\frac{n+1}{n}\left( 1-S_{p} \right) \right)+\left( 1-\left( 1-p \right)^{n} \right)(\frac{n+1}{n}S_{ep}(t,n)+\frac{1}{n}(1-S_{ep}(t,n)))$.

Thus, for all $n$, the per-subject expected number of tests $\mathbb{E[}T]$ can be written as:

$\mathbb{E[}T]=\left\{ \begin{aligned} 1, n=1 \\ \frac{1}{n}+S_{ep}(t,n)-\left( S_{ep}(t,n)+S_{p}-1 \right)\left( 1-p \right)^{n}, n>1 \end{aligned} \right.$.

The per-subject expected number of true positives $\mathbb{E[}TP]$ measures the test effectiveness and is calculated by:

$\mathbb{E[}TP]=\left\{ \begin{aligned} S_{ei}(t)p, n=1 \\ S_{ed}(t,n)p, n>1 \end{aligned} \right.$.

Finally, $CPR(n,t)$ is given by:

$$CPR\left( n,t \right)=\frac{\mathbb{E}\left[ T \right]}{\mathbb{E[}TP]}=\left\{ \begin{aligned} \frac{1}{S_{ei}(t)p}, &n=1 \\ \frac{1}{S_{ed}(t,n)p}\left\{ \frac{1}{n}+S_{ep}(t,n)-\left( S_{ep}(t,n)+S_{p}-1 \right)\left( 1-p \right)^{n} \right\}, & n>1 \end{aligned}. \right.$$

Although it’s difficult to get the close form expression of $S_{ep}(t,n)$, $S_{ed}(t,n)$, and $S_{ei}(t)$, we can build regression models to obtain numerical results of these functions. These results are shown in Figure 2.

**Epidemic Model**

The epidemic model captures the epidemic dynamics and the effects of population screening over time. In our setting, we divided individuals into five compartments, including the Susceptible (S), Infected (I), Isolated (Q), Self- Isolated (SQ), and Recovered (R) compartments. Susceptible individuals $S_{t}$ are infected through contact with unidentified infected individuals $I_{t}$. The number of new infections is given by $S_{t+1}-S_{t}=-\frac{\beta S_{t}I_{t}}{N}$, where $\beta$ denotes the transmission rate. Unlike traditional SIR model, the epidemic dynamics evolve as individual-level viral load trajectories and the test-isolation program. Parameters used in the epidemic transmission model are summarized in Table S2. Parameters of individual-level viral load trajectories are summarized in Table S1.

As PCR tests has turnaround time, let $T_{delay}$ be the delay of reporting. During time interval t, individuals who get the delayed positive results (tested $T_{delay}$ days ago) will be isolated. We assume people who get positive results will be confirmed to exclude false positive results. Thus, only infected patients will be isolated. In the pooled PCR testing setting, the pooled sample has a positive result only when the average viral load in this pool is higher than the limit of detection (LOD). A positive result for the individual sample requires that the viral load of individual sample is higher than LOD. The number of isolated individuals decreases as patients recover. The change of isolated population is calculated by:

$$Q_{t+1}-Q_{t}=\sum_{i\in I_{t}} 1\left( i test positive at \left( t-T_{lead} \right) \right)-\sum_{i\in Q_{t}} 1\left( t>t_{inf}^{i}+t_{inc}^{i}+t_{w}^{i} for the first time \right)$$

We also assume that infected individuals will isolate themselves after developing symptoms. The number of self-isolated individuals decreases as patients recover. Although all individuals are assigned an incubation period $t_{inc}^{i}$, only symptomatic individuals will have symptom onset. The fraction of symptomatic individuals in new infected individuals is $p_{s}$. The change of self-isolated population is calculated by:

$$SQ_{t+1}-SQ_{t}=p_{s}\sum_{i\in I_{t}} 1\left( t>t_{inf}^{i}+t_{inc}^{i} for the first time \right)-\sum_{i\in{SQ}_{t}} 1\left( t>t_{inf}^{i}+t_{inc}^{i}+t_{w}^{i} for the first time \right)$$

Finally, all infected individuals will recover according to their viral load trajectories. The increase of recovered population is given by:

$$R_{t+1}-R_{t}=\sum_{i\in I_{t}\cup Q_{t}\cup SQ_{t}} 1\left( t>t_{inf}^{i}+t_{inc}^{i}+t_{w}^{i} for the first time \right)$$

**Table S2. Description of parameters used in the epidemic transmission model.**

| **Parameter** | **Description** | **Value** |
| --- | --- | --- |
| $R_{0}$ | Basic reproductive number | 2.5 (fixed) |
| $\beta$ | Transmission rate | 0.12 (fixed) |
| $I_{0}$ | Initial number of infected individuals | 50 (fixed) |
| N | Population size | 100,000 (fixed) |
| $p_{s}$ | The fraction of symptomatic infected individuals | 0.3 (fixed) |
| $T_{end}$ | The longest run time of the simulation | 365 days |

**Cost analysis**

The cost breakdown analysis of PCR tests is based on reference 17. The cost of PCR tests can be categorized into two parts: (1) reagents/consumables costs and (2) labor costs. The cost of employing technicians to do RT-PCR tests was $36.50 per hour^17^. Antigen screening only costs $10 per testing kits^30^. All cost data come from the US data.

The categorized costs are allocated to per test or per sample according to the practice (Table S3). Then we can evaluate the cost of four screening schemes based on simulation results and categorized costs. We assume the unit costs of materials and labor maintained constant throughout the simulation.

**Table S3. Description of parameters used in the cost analysis.**

| **Category** | **Unit** | **Value** |
| --- | --- | --- |
| **PCR tests** | | |
| Cost of reagents and consumables for RNA extraction | $ per test | 9.18 |
| Cost of reagents and consumables for RT-PCR | $ per test | 5.43 |
| Labor cost of RNA extraction | $ per test | 1.30 |
| Labor cost of RT-PCR | $ per test | 0.65 |
| Labor cost of set-up for original pools | $ per sample | 0.24 |
| Labor cost of reporting | $ per sample | 0.91 |
| **Antigen tests** | | |
| Cost of antigen test kit | $ per test | 10.00 |
